# Confirmation of factors that influence antiretroviral regimen change and the subsequent patient outcomes at a Regional Hospital in rural KwaZulu-Natal

**DOI:** 10.4102/phcfm.v8i1.1171

**Published:** 2016-10-31

**Authors:** Vereesha Soorju, Panjasaram Naidoo

**Affiliations:** 1 Madadeni Hospital, School of Health Sciences, University of KwaZulu-Natal, South Africa

## Abstract

**Background:**

Treatment failure (TF) and adverse drug reactions (ADRs) are the main indications for antiretroviral therapy (ART) regimen change. Identification of factors influencing regimen change and subsequent health outcomes of patients after regimen change is essential in providing a sustainable and effective antiretroviral roll-out campaign.

**Aim:**

To confirm the factors that influence antiretroviral regimen change and to evaluate patient outcomes post regimen change.

**Methods:**

A retrospective chart analysis of 269 HIV-infected non-pregnant patients (age >18 years), who underwent an antiretroviral (ARV) regimen change and were followed up for approximately one year since initiation, was undertaken at a Provincial Hospital ARV Clinic in KwaZulu-Natal, from January 2008 to December 2012.

**Results:**

Of the 269 patients, there were 200 females (75%). Most patients were between the ages 30 and 44 (57.6%). Only five patients had coexisting tuberculosis (TB) infection (2%). The most common first-line ART regimen to be changed was stavudine (D4T)/lamivudine(3TC)/ efavirenz(EFV) *n* = 111(41%). The most common regimen patients were changed to was tenofovir (TDF)/3TC/EFV *n* = 89(33%). Stavudine was the most commonly substituted drug (35.5%). Lipodystrophy was the most common ADR (56.8%). ADR was the indication for ART regimen change in 175 patients (65%), whilst TF accounted for ART regimen change in 94 patients (35%). Immunological success (CD4 counts) was shown after regimen change (374.21 ± 243.16 vs. 456.09 ± 250.07, CI: 0.95, *p* < 0.001). Undetectable viral loads were measured in 172/205 (83.9%) patients post regimen change.

**Conclusion:**

ADRs were the main cause for antiretroviral regimen change. Stavudine was the most substituted drug with lipodystrophy being the most common side effect. Coexisting TB infection did not influence regimen change. Immunological and virological success was shown after regimen modification.

## Introduction

South Africa has the highest number of HIV-infected people worldwide with an estimated 5.6 million people living with HIV.^1^ A strategic report in 2005 reported an antenatal sero-prevalence of 22%, which rose to 30% in 2014 reflecting an increase in the number of HIV infection in the population.^1^ The antiretroviral roll-out campaign in South Africa has gained much momentum in the last decade. In April 2010, treatment was offered in fewer than 500 facilities – by the end of 2013 that number had risen to over 3500, and the number of people receiving antiretroviral therapy (ART), from less than 1 million to greater than 2 million.^1^

Despite the success in access and distribution of ART, several challenges exist with respect to adverse drug reactions (ADRs) and treatment failure (TF), which are indications for ART regimen change.^2^ These two factors are intimately related as an ADR may lead to poor adherence, which ultimately results in TF.^2^ During the period 2007–2011, drug-related toxicity from first-line regimens was found to be a major contributing factor to ART regimen change in the South African context.^3^ ART regimen change can be challenging when drug availability, cost and acceptable combinations are limited by their pharmacokinetic and toxicity profiles, thus allowing for only a certain number and class of drug substitutions to occur. Personal determinants of poor adherence have been investigated, which included factors, such as stigma, discrimination, depression and alcohol, that would negatively affect patients’ outcomes.^2^

The resurgence of tuberculosis (TB) has largely paralleled the HIV pandemic with more than 50% of HIV-infected patients developing TB in their lifetime. Highly active antiretroviral therapy (HAART) has led to a decrease in HIV-associated TB by more than 80%.^4^ Despite the reported success, several drug interactions exist between ART and anti-TB therapy consequently affecting the pharmacokinetic properties of ART, often resulting in reduced plasma levels of both non-nucleoside reverse transcriptase inhibitors and protease inhibitors (PI).^4,5^ This may contribute to TF, thus influencing ART regimen change.

Frequent monitoring enables the diagnosis of virological failure before the development of drug resistance mutations, which would ultimately lead to TF and allow for possible viral transmission.^6^

Monitoring the immunological (CD4) and virological response (viral load[VL]) to ART regimen change is essential in determining the efficacy of regimen changes and individual drug substitutions.

Hence, the aim of this study was to confirm and quantify the factors that influence ART regimen change and to evaluate the health outcomes of the patients post regimen change using CD4 and VL as markers.

## Methods

A retrospective chart analysis of 269 HIV-infected patients who underwent an ART regimen change between January 2008 and December 2012 and who were followed up for approximately one year after an ART regimen change at a rural Provincial Hospital ARV Clinic, situated in Newcastle, KwaZulu-Natal, South Africa, was undertaken. Patients who had a regimen change were sampled in chronological order. Data were manually retrieved from outpatient files.

Patients under 18 years of age and pregnant patients were excluded from the data analysis, the latter being excluded because the Madadeni Hospital ARV Clinic does not render a maternity and paediatric service. Variables included in the data analysis were age, gender, first-line ART regimen, coexisting TB infection, modified ART regimen, ADRs, drug substitutions, indication for regimen change and time to regimen change.

Outcome measures included the viral load and CD4 counts prior to ART regimen change and at six months follow up after ART regimen change. An undetectable viral load was given a numerical value of 39 for purposes of statistical analysis.

Descriptive statistics, using means and standard deviations for continuous variables, and frequencies for categorical variables, were used to report sample characteristics. A Student’s *t*-test was used to compare continuous variables. A *p* < 0.05 was considered statistically significant. SPSS version 23 software (Chicago, IL, USA) was used for the data analysis.

## Ethical considerations

Gatekeepers permission was given by the KwaZulu-Natal Department of Health Research and Development Committee with the study given full ethical approval from the Biomedical Research Ethics Committee of the University of KwaZulu-Natal (BREC No: 206/15).

## Results

Of the 269 patients who underwent ART regimen change, there were 200 females (75%) and 69 males (25%). Most patients were between the ages 30 and 44 (57%); >45 years (25%) and 18–29 years (16%). Only five patients had coexisting TB infection (2%) (Table 1).

**TABLE 1:** Socio-demographic and immunological profile (entire cohort).

Variables	*n* (%)
Gender	
Females	200 (74.3)
Males	69 (25.7)
**Age**	
18–29 years	43 (16)
30–44 years	159 (59.1)
>45 years	67 (24.9)
**Time to change (months)**	34.6
**Coexisting TB infection**	5(2)
**Indication for ART change**	
Adverse drug reaction	175 (65)
Treatment failure	94 (35)
**Preceding CD4 count (cells/mm^3^) (mean ± s.d.)**	374.21 ± 243.16
**Preceding viral load (copies/mL)**	
Undetectable	168 (62.5)
40–499	5 (1.9)
>1000	96 (35.7)

*Source:* The contents of Tables 1–5 are from our data analysis from the data collected from the ARV Clinic at Madadeni Hospital

ART, antiretroviral therapy; TB, tuberculosis.

The most common first-line ART regimens to be changed were stavudine (D4T)/lamivudine (3TC)/ efavirenz (EFV) *n* = 111(41%), D4T/3TC/ nevirapine (NVP) *n* = 100(37%) and TDF/3TC/EFV *n* = 26(10%).

The most common regimens that patients changed to included TDF/3TC/EFV *n* = 89(33%), TDF/3TC/NVP *n* = 75(28%) and TDF/NVP/LPV *n* = 55(20%) (Tables 2 and 3). The three most common drug substitutions were D4T *n* = 144(35.5%), EFV *n* = 34(12.6%) and NVP *n* = 34(12.6%).

**TABLE 2:** Frequency of initial antiretroviral therapy (ART) regimen.

Initial ART regimen	*N* (%)
AZT/3TC/EFV	5 (1.9)
AZT/3TC/LPVr	1 (0.4)
AZT/3TC/NVP	3 (1.1)
AZT/ABC/LPVr	1 (0.4)
AZT/DDI/LPVr	1 (0.4)
D4T/3TC/EFV	111 (41.3)
D4T/3TC/LPVr	1 (0.4)
D4T/3TC/NVP	100 (37.2)
TDF/3TC/EFV	26 (9.7)
TDF/3TC/NVP	20 (7.4)

*Source:* The contents of Tables 1–5 are from our data analysis from the data collected from the ARV Clinic at Madadeni Hospital

ART, antiretroviral therapy; D4T, stavudine; 3TC, lamivudine; EFV, efavirenz; NVP, nevirapine; AZT, zidovudine; DDI, didanosine; TDF, tenofovir; ABC, abacavir; LPVr, lopinavir/ritonavir; *N*, number.

**TABLE 3:** Frequency of modified antiretroviral therapy (ART) regimen.

Modified ART regimen	*N* (%)
ABC/3TC/EFV	1 (0.4)
ABC/3TC/LPVr	5 (1.9)
AZT/3TC/EFV	1 (0.4)
AZT/3TC/LPVr	40 (14.9)
AZT/3TC/NVP	2 (0.7)
D4T/3TC/LPVr	1 (0.4)
TDF/3TC/EFV	89 (33.1)
TDF/3TC/LPVr	55 (20.4)
TDF/3TC/NVP	75 (27.9)

*Source:* The contents of Tables 1–5 are from our data analysis from the data collected from the ARV Clinic at Madadeni Hospital

ART, antiretroviral therapy; D4T, stavudine; 3TC, lamivudine; EFV, efavirenz; NVP, nevirapine; AZT, zidovudine; DDI, didanosine; TDF, tenofovir; ABC, abacavir; LPVr, lopinavir/ritonavir; *N*, number.

The most common ADR was lipodystrophy (56.8%) (Table 4).

**TABLE 4:** Frequency of adverse drug reactions in entire cohort (*n* = 269).

ADRs	3TC	AZT	D4T	EFV	NVP	Total
Lipodystrophy	0	2	150	0	0	152
Anaemia	0	2	0	0	0	2
Peripheral neuropathy	0	0	7	0	0	7
Lactic acidosis	0	0	1	0	0	1
Skin reaction	0	0	0	0	1	1
Steven Johnson syndrome	0	0	0	0	3	1
CNS effects	0	0	0	2	0	2

**Total**	**0**	**4**	**158**	**2**	**4**	**168**

*Source:* The contents of Tables 1–5 are from our data analysis from the data collected from the ARV Clinic at Madadeni Hospital

ADRs, adverse drug reactions; 3TC, lamivudine; AZT, zidovudine; D4T, stavudine; EFV, efavirenz; NVP, nevirapine; CNS, central nervous system.

ADR was the indication for ART regimen change in 175 patients (65%) and TF reason for change in 94 patients (35%).

There was a significant difference in the time to regimen change in the TF and ADR groups (30.77 vs. 36.72 months), *p* < 0.002. The mean follow up time for CD4 and viral load counts was 14.31 months.

There was a significant increase in the mean CD4 counts post regimen change in the entire cohort (374.21 ± 243.16 [pre] vs. 456.09 ± 250.07 [post]), *p* < 0.001. There was a significant difference between the CD4 counts post regimen change in the TF and ADR groups (290.65 ± 180.60 vs. 543.90 ± 233.56 cells/mL), *p* < 0.001) (Table 5).

**TABLE 5:** Outcome of ART regimen change – CD4 count data analysis between TF and ADR groups.

Variable	Preceding CD4 (cells/mm^3^) Mean ± s.d.	Post change CD4 (cells/mm^3^) Mean ± s.d.	*p*
Treatment failure	174.28 ± 144.37	290.66 ± 186.60	*p* < 0.001
Adverse drug reaction	500.16 ± 212.375	543.90 ± 233.56	*p* = 0.003

**Total cohort**	**374.21 ± 243.16**	**456.09 ± 250.07**	***p* < 0.001**

*Source:* The contents of Tables 1–5 are from our data analysis from the data collected from the ARV Clinic at Madadeni Hospital

s.d., standard deviation.

Pre-ART regimen change viral load data are shown in Table 1. Sixty-four patients had missing post regimen change viral load data. Of the remaining 205 patients with post regimen change viral load data, 172/205 (83.9%) patients had undetectable viral loads after regimen change, 40–499 copies/mL: 11(5.4%) and >1000 copies/mL: 18(8.8%).

## Discussion

### Main reasons for antiretroviral therapy regimen change

The main indication for ART regimen change globally includes TF and ADR.^3^ In this study, ADR (65%) was the most frequent indication for ART regimen change. The high frequency of ADR in this study may be attributed to the high number of first-line regimens containing stavudine, which accounted for the highest number of drug-specific toxicity, namely lipodystrophy. This finding is consistent with a study done by Lima et al. who have shown that 88.5% of ART regimen changes were because of ADR requiring drug substitutions.^7^ In another study, Kumarasamy et al. have demonstrated that the most common reason for changing ART regimens was ADR (64%),^8^ whereas Sidavasan et al. have shown drug toxicity to account for 27% of their ART regimen changes.^9^ Locally, Orell et al. have shown that only 1.7% of patients changed to a second-line regimen because of drug toxicity,^10^ but this study was conducted between 2002 and 2005 at which time second-line regimens were not routinely available in South Africa. Isolated drug substitutions were more commonly practised at the time.

TF accounted for 35% of ART regimen change in this study. The low prevalence of TF may be as a result of improved levels of patient adherence because of robust counselling and patient education. Reasons cited in studies for virological failure include poor adherence, genetic factors and drug interactions, which lead to reduced antiretroviral (ARV) concentrations and transmitted drug resistance.^11^ Locally, Dube et al. had shown that TF was the second most common reason for regimen change.^3^

TF has far more serious implications when switching to a second-line regimen because of the inclusion of PI. The major pitfalls of a change to a second-line PI-based regimen include complex and frequent dosing, which may lead to poor adherence.

A study conducted by Paterson et al. of patients on PI-based regimens showed that adherence was significantly associated with successful virologic outcome (*p* < 0.001) and increase in CD4 lymphocyte count (*p* < 0.006). Virologic failure was documented in 22% of patients with adherence of 95% or greater, 61% of those with 80–95% adherence, and 80% of those with less than 80% adherence.^12^

### Coexisting tuberculosis infection and antiretroviral therapy regimen change

In this study, coexisting TB infection occurred in only 2% of the study population and was a non-contributory factor influencing ART regimen change. Lima et al. have shown that drug interactions between ART and anti-TB drugs accounted for only 3.8% of ART regimen changes.^7^

In contrast, a study by Dean et al., which involved HIV and TB co-infected patients, presented a total of 167 adverse events that were recorded in 99 (54%) of the 183 patients for whom data on therapy were available. Adverse events led to cessation or interruption of either their TB or HIV therapy in 63 (34%) of the 183 patients. The most common side effects noted were peripheral neuropathy, rash, gastrointestinal, hepatitis and neurological events.^13^

Dean et al. also showed that peripheral neuropathy was the most frequently documented adverse event (21%). Twenty-two of these 39 patients (56%) were taking ARV concomitantly. This adverse event led to an interruption of TB/HIV therapy in 19 cases.^9^ Sixteen (27%) of 56 patients who received isoniazid and stavudine concomitantly developed peripheral neuropathy compared with 21 of 124 (17%) patients prescribed isoniazid without stavudine.^13^ In our study, the low prevalence of TB co-infection and the use of concomitant anti-TB therapy did not impact on drug–drug interactions with stavudine, which was the most common agent used in the entire cohort.

There are various reasons why patients may develop peripheral neuropathy (advanced HIV disease, TB, anti-tuberculous or ART and nutritional deficiencies); the study by Dean et al. indicated that this side effect was mainly observed in patients prescribed isoniazid with antiretrovirals known to be neurotoxic.^13^ The study stated an observed rate that was higher than that reported in HIV-infected patients without TB who were prescribed stavudine and didanosine (8%) and HIV-negative patients prescribed isoniazid for TB (0.15%). However, other studies reported a greatly increased incidence of peripheral neuropathy (55%) for HIV-infected patients prescribed isoniazid and stavudine concomitantly.^13^

Although the study by Dean et al. showed that a higher percentage of patients receiving this combination developed peripheral neuropathy compared with patients receiving isoniazid alone, this did not attain statistical significance. Given that a high proportion of these patients had either their TB or HIV medication changed in an attempt to alleviate the symptoms, caution should be exercised when prescribing multiple agents known to cause nerve damage in high-risk patients.^13^

Despite the low incidence of reported ART–anti-TB therapy drug interactions in this study, clinicians should still be aware of the potential for toxicity and clinically monitor for side effects.^13^

## Most common adverse drug reactions

ADRs were most commonly caused by stavudine (D4T) (35.5%) with associated lipodystrophy being the most common side effect in this study. Other studies have shown stavudine toxicity accounted for 76% of ART regimen changes.^9^

A local study by Dube et al. has established that 34% of patients had changed from their initial ARV regimen. This study revealed that of the patients who had changed regimen because of ARV-related toxicity, 76.1% of patients changed from a stavudine-based regimen to a zidovudine-based regimen.^3^

In addition, the study by Dube et al. depicted that the most common adverse effects experienced by patients were polyneuropathy (24%), lipodystrophy (23.9%), neuropathy (10%) and suspected lactic acidosis (3.8%).^3^

However, Lima et al. have shown that zidovudine (AZT) was the highest substituted drug (42.4%) with anaemia as the most common adverse side effect.^7^ The significant toxicity associated with D4T and especially after cumulative exposure has led to the removal of D4T as part of first-line ARV treatment according to the National Department of Health (NDOH) guidelines as of 2015.^14^ A fixed dose combination (FDC) of TDF/FTC/EFV has been implemented as part of first-line treatment since 2013. The benefits of phasing out of D4T include the exclusion of a significantly toxic drug, a reduction in stigma associated with lipodystrophy and lipoatrophy, and a decline in mortality because of lactic acidosis.^14^ Our findings have shown that 99% of patients were changed to a non-D4T-containing regimen in keeping with NDOH guidelines.

The inability of patients to manage the side effects associated with ARVs may result in poor adherence of treatment regimens. Management of HIV is a multidisciplinary approach. It is imperative to screen patients for signs and symptoms of adverse effects or drug toxicity. Pharmacists play a key role in ensuring the safe, rational and appropriate use of medicine, as well as provision of pharmaceutical care by monitoring patient outcomes.

The mean time to ART regimen change was 34.5 months with a significantly higher duration in the ADR group. This may be attributed to the high frequency of lipodystrophy in this study population, which fares as a tolerable side effect thus delaying the urgency for ART regimen change.

Although Dube et al. have indicated that the average time to regimen change was 14.9 months,^3^ Sivadasan et al. have shown that serious drug toxicities (skin reactions) occur as early as from 2 weeks up to 42 weeks (lactic acidosis) necessitating earlier ART regimen change.^9^

## Monitoring of outcomes of antiretroviral therapy regimen change

The outcome of ART regimen change to a second-line regimen can be monitored clinically (WHO AIDS clinical staging), immunologically (CD4 counts) and virologically (viral load). We chose to monitor the VL and CD4 at 6 months post regimen change to assess the therapeutic outcome. In this study, the mean follow up CD4 and VL counts were done at 14.31 months. The significant delay may be attributed to poor socio-economic status and lack of transport infrastructure especially in the rural setting.

There was a statistically significant increase in the CD4 count following regimen change, suggesting immunological success in both TF and ADR groups. The significant increase in the post regimen change CD4 count in the ADR group may be attributed to poor patient adherence when experiencing intolerable side effects. Virological success was achieved in 83.9% of patients who had undetectable viral load after changing regimen. The WHO has proposed that 70% of patients should achieve virological suppression (<400 copies/mL) after 6 months of ART.^15^ Another study had shown a treatment success rate of 82% following a change to a second-line regimen at 1 year follow up.^16^ In our study, the mean gain in CD4 count post regimen change at 14.31 months was 82 cells/mm^3^, which compares favourably with local studies from Johannesburg that reported a mean increase in CD4 count of 133 cell/mm^3^ following regimen change.^17^ Because of poor laboratory support and follow up, viral loads were not routinely measured in 64/269 patients (24%). In a study from Khayelitsha, South Africa, Boulle et al. have shown that viral load monitoring is an essential tool in the early identification of TF and for monitoring patient adherence even in the resource constrained setting.^18^

Frequent monitoring enables the diagnosis of virological failure before the development of drug resistance mutations, which would ultimately lead to TF and allow for possible viral transmission.^6^ In the absence of virological monitoring, immunological monitoring by CD4 count change is recommended. However, CD4 testing has a poor accuracy and low positive predictive value for diagnosing TF. The limitations associated with immunological monitoring could lead to patients being diagnosed very late or misdiagnosed completely, with the result that patients can be kept on a failing regimen or switched unnecessarily; hence, routine virological monitoring remains the gold standard.^6^

## Limitations

Incomplete sets of data, particularly the lack of viral load data in the entire cohort, precluded an accurate assessment of virological response rates following ART regimen change. The rural-based site of the study may not be a representative of the HIV-infected patient population at large, where poor socio-economic status delays to hospital care and poor follow up of CD4 and VL are more prevalent.

## Conclusion and recommendations

This study has confirmed that both ADR and TF are in fact reasons for ART regimen change in patients attending this rural ARV Clinic with ADR being a more common reason for change than TF. The most commonly implicated drug was stavudine (D4T) with its associated toxicity, namely lipodystrophy being the most common side effect. TB was not a determining factor influencing ART regimen change. The change of regimen had resulted in significant improvements in both post ART regimen immunological and virological success rates. This highlights the importance of stringent patient follow up, early identification of ADR and timeous switching to an alternative regimen.

Readily available access to ARV clinics, routine monitoring of patients and appropriate patient counselling constitute key elements in ensuring favourable patient outcomes.
